# Novel Genetic Rearrangements in Hepatitis B Virus: Complex Structural Variations and Structural Variation Polymorphisms

**DOI:** 10.3390/v13030473

**Published:** 2021-03-12

**Authors:** Kei Fujiwara

**Affiliations:** Department of Gastroenterology and Metabolism, Nagoya City University Graduate School of Medical Sciences, Nagoya 467-8601, Japan; keifuji@med.nagoya-cu.ac.jp; Tel.: +81-52-853-8211; Fax.: +81-52-852-0952

**Keywords:** hepatitis B virus, genome, complex structural variation, genetic rearrangement, bioinformatics, insertional motif, hepatocyte nuclear factor 1 binding site, pairwise/multiple alignment, mutation, structural variation polymorphisms

## Abstract

Chronic hepatitis B virus (HBV) causes serious clinical problems, such as liver cirrhosis and hepatocellular carcinoma. Current antiviral treatments suppress HBV; however, the clinical cure rate remains low. Basic research on HBV is indispensable to eradicate and cure HBV. Genetic alterations are defined by nucleotide substitutions and canonical forms of structural variations (SVs), such as insertion, deletion and duplication. Additionally, genetic changes inconsistent with the canonical forms have been reported, and these have been termed complex SVs. Detailed analyses of HBV using bioinformatical applications have detected complex SVs in HBV genomes. Sequence gaps and low sequence similarity have been observed in the region containing complex SVs. Additionally, insertional motif sequences have been observed in HBV strains with complex SVs. Following the analyses of complex SVs in the HBV genome, the role of SVs in the genetic diversity of orthohepadnavirus has been investigated. SV polymorphisms have been detected in comparisons of several species of orthohepadnaviruses. As mentioned, complex SVs are composed of multiple SVs. On the contrary, SV polymorphisms are observed as insertions of different SVs. Up to a certain point, nucleotide substitutions cause genetic differences. However, at some point, the nucleotide sequences are split into several particular patterns. These SVs have been observed as polymorphic changes. Different species of orthohepadnaviruses possess SVs which are unique and specific to a certain host of the virus. Studies have shown that SVs play an important role in the HBV genome. Further studies are required to elucidate their virologic and clinical roles.

## 1. Introduction

Chronic hepatitis B virus (HBV) infection is among the causes of liver cirrhosis and hepatocellular carcinoma (HCC). It has been estimated that 248 million people are positive for hepatitis B surface antigen [1], and HBV is among the most serious infectious diseases worldwide.

HBV research began with the discovery of the Australian antigen in 1965 [2]. The genetic sequences of HBV were determined in 1979 [3,4,5]. Okamoto and colleagues [6] defined the HBV genotype as nucleotide differences of greater than 8%, and classified HBV genotypes A to D. The HBV genotype was later confirmed by phylogenetic analysis [7]. Presently, eight genotypes (A to H) have been reported, and they reflect different geographical distribution [8,9]. In addition, two new genotypes (I and J) have been reported [10,11].

Nucleotide substitution plays an important role in HBV genetic changes. As mentioned above, the HBV genotype is defined by nucleotide substitution of greater than 8%. Approximately 256 nucleotide substitutions in the entire HBV genome separate each genotype.

In addition, unique point mutations have been reported. The pre-core (PC) mutation G1896A was reported in 1989. The mutation caused a stop codon in the gene for the hepatitis B e antigen (HBeAg) and was detected in a patient who seroconverted to anti-HBeAg antibodies [12]. In an analysis of HBV mutations related to HBeAg seroconversion, core promoter (CP) mutations (A1762T/G1764A) were also found [13].

Recombination is another important genetic change in HBV [14,15,16,17,18,19]. The differences between HBV genotype B in Japan and Taiwan were explained by intergenotypic recombination. HBV genotype B in Taiwan consisted of genotype B and C recombination [20]. Genetic alteration of HBV has been explained by single nucleotide changes, such as nucleotide substitutions, CP and PC mutations and recombination.

## 2. Complex Structural Variation in the Cellular Genomes of Humans and Mice

Basically, structural variation (SV) in DNA genomes is defined by canonical forms, such as insertion, deletion, duplication and inversion. Recent studies have clarified novel variants that cannot be included among those canonical forms [21,22]. The non-canonical patterns of genetic alterations are called complex SVs [23,24,25,26,27,28]. Quinlan and Hall [24] explained that complex SVs are composed of multiple breakpoints whose origin cannot be explained by single end-joining or DNA exchange events. In addition, Yalcin and colleagues [25] defined complex SVs as two or more SVs co-occurring at the same locus. Complex SVs can modify the normal regulation of the genome by changing an enhancer, promoter, or repressor sequence into a new sequence, and can affect biological and clinical function [24].

## 3. Complex SVs Observed in the HBV Genome

**Figure 1:**
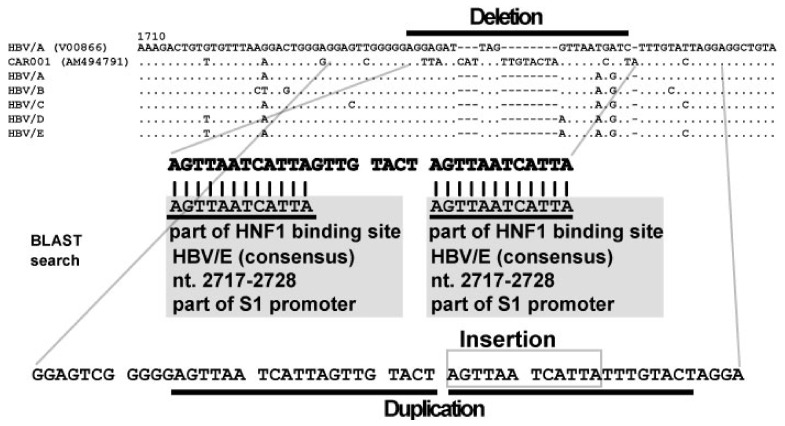
The pattern of complex structural variations (SVs) observed in CAR001 (AM494791) is shown. Nucleotide alignments of HBV genotype A (HBV/A)) (V00866) reference sequence and consensus reference sequences of HBV/A to HBV/E are shown with CAR001. Deletion of normal basic CP (BCP) sequences was observed, and part of the Pre-S1 promoter containing part of the hepatocyte nuclear factor 1 (HNF1) binding site was inserted. Internal duplication was also observed. HB X open reading frame (ORF) was involved. The complex SVs caused putative HBV X protein truncated 22 amino acids at the C-terminal. For the origin of the HBV strains, 39 were from Asia, followed by 9 from Europe, 9 from the Middle East, 7 from Africa and 6 from America, respectively. Detailed information of 70 strains with complex SVs are shown in additional files 1 and 2 of Reference [31]. Twenty-four were isolated from serum/plasma. For the reference sequences, we analyzed consensus reference sequences of HBV/A to E using CLUSTAL W. To determine these consensus sequences, 150, 40, 168, 79 and 38 complete genome sequences of HBV/A, HBV/B, HBV/C, HBV/D and HBV/E, respectively, were used, as described in Reference [30].

Detailed genetic analysis of an HBV strain clarified a novel genetic change [29]. The combination of an insertion, a deletion and a duplication was observed in the HBV genome. Additionally, these SVs were observed in the same locus. This pattern of genetic change in the HBV genome had not been reported previously. In 2005, the concept of the observed genetic event had not yet been defined. Subsequently, the concept of complex SVs was established by studying genome variations of model species [23,24,25,26,27,28]. The definition of complex SVs in the human and mouse genomes were used to describe complex SVs in HBV. Presently, 70 HBV strains isolated from Asia, Europe, the Middle East, Africa and America have been confirmed to contain complex SVs, and their complex genetic structures have been clarified [30,31]. The proportions of HBV genotype C, B and D were much higher than other genotypes, and clinically, 11 strains with complex SVs were from patients with HCC [31]. A typical HBV strain with complex SVs is shown in Figure 1. The replication competence of HBV strains with complex SVs has been shown in some strains in previous studies. For example, the longitudinal time course of one strain was analyzed. At first, wild-type HBV genome was predominant, then the proportion of the strain with complex SVs was increased and became the major strain [29].

## 4. Bioinformatical Analysis for the Detection of Complex SVs in HBV

Complex SVs are defined by SVs with multiple breakpoints and are composed of combinations of insertions, inversions, deletions and duplications [24,25]. In initial bioinformatics analysis, candidate areas in genetic sequences were searched by pairwise or multiple-alignment analysis using the CLUSTAL W [32], MAFFT [33], or T-coffee [34] program. Investigators identified partial sequences with low sequence identities with and without sequence gaps, and then NCBI BLAST [35] search for unique genetic sequence fragments was performed. A previous study showed that 80% of complex SVs in HBV had sequence gaps in pairwise or multiple sequence alignment analyses [31]. Therefore, sequence gaps are an important marker to identify complex SVs. Subsequently, the architecture of complex SVs in humans and mice [24,25] was considered as a reference, and detailed analysis with visual inspection was performed. Unique insertional motif sequences were identified in the first report of complex SVs in HBV [30]. The first such sequence discovered was the HNF1 binding site. For example, the HBV strain with complex SVs shown in Figure 1 contains part of the HNF1 binding site as an insertional motif. The Pre-S1 promoter contains HBV HNF1 binding site, and the sequence is conserved in many HBV strains. An insertion of unknown origin (GAAGAGCTCAAGCTTTGC) (subsequently named X-1 as described below) was discovered as the second insertional motif. When a low sequence similarity between the query sequence and reference sequence was observed in visual inspection, the origin of the unique query sequence was searched using NCBI BLAST [35]. HBV (taxid:10407) was selected in the “Organism” field of the “Choose Search Set” option. The results of the BLAST search suggested the structure of the sequence fragment and additional candidate strains.

## 5. Points Where Complex SVs Are Observed in the HBV Genome

The most frequent locations of complex SVs in HBV were investigated to determine whether the novel genetic rearrangements are equally distributed in the HBV genome. The data showed that 94.3% (66/70) of complex SVs were observed between nt 1500 and 2000 of the HBV genome (V00866). This area corresponded to the X ORF, pre-core/core ORF. Additionally, important regulatory regions for HBV transcription, such as core upstream regulatory sequences/BCP, enhancer II and direct repeat (DR)1/DR2, were included in this area. In addition, the region nt 1500–2000 of the HBV genome corresponds to frequent recombination points. Araujo et al. [19] reported that specific favored sites of recombination were detected within the range nt 1700–2000, which lies in the vicinity of the DR1 region. The frequency of recombination within nt 1700–2000 displayed a statistically significant difference in comparison with the remaining genome. An experimental study by Hino et al. [36] showed that a fragment containing the DR1 region covering nt 1824–1834 is indispensable for the enhancement of in vitro recombination. In addition, the region is prone to recombination events, as it includes the 5′ and 3′ ends of the HBV double-stranded linear (dsl) DNA, which can integrate into host chromosomal DNA at double-strand DNA breaks [37]. Further, the most frequent breakpoints of HBV integration have been observed in the C-terminal region of HBx gene around the DR1 sequence (1817–1836) corresponding to the end of HBV dsl DNA [38]. In addition, recent integration analysis showed that viral-host breakpoints were more prone to being located at the 3′-end of the X gene, particularly with the sequences from nt 1400 to 1900 [39]. Frequent breakpoints of HBV integration correspond to the locations of complex SVs in some HBV strains. Therefore, it is possible that some of the insertional motifs in complex SVs are derived from the host genome.

## 6. Discovery of Insertional Motif Sequences in Complex SVs in HBV

The existence of unique insertional motif sequences in complex SVs was clarified during the analyses. The HNF1 binding site was the first motif sequence identified. “AGTTAATCATTAC” is the consensus nucleotide sequence of the HNF1 binding site, and is located in the pre-S1 promoter region. HNF1 is a transcriptional factor that binds to a specific DNA sequence. [40,41,42,43,44]. Mutations that disrupt the HNF1 binding tend to severely impair the ability of promoters to direct liver-specific transcription [43]. In HBV, the HNF1 binding site is involved in the pre-S1 promoter, and the pre-S1 promoter controls transcription of pre-S mRNA [45,46,47,48]. Transcriptional activity of the pre-S1 promoter is up-regulated by the HNF1 binding site [49]. Studies have shown that the transcriptional activity of pre-genomic RNA was also up-regulated when simple insertion of the HNF1 binding site into BCP occurred [50,51]. In the viral genome analyses of HBV patients with post-organ transplantation or HBV patients with fulminant hepatic failure, HNF1 binding site insertion into BCP was observed [50,51,52,53]. One study has shown that the role of BCP mutations (A1762T/G1764A) is to compose a new HNF1 binding site in the BCP [54]. From these data, it is speculated that the creation of a new HNF1 binding site in the BCP is selected in the HBV genome.

The second insertional motif sequence was “GAAGAGCTCAAGCTTTCC (X-1)”. The origin of X-1 was not identified by the BLAST search. Interestingly, a sequence complementary to X-1 was also detected as an insertional motif [30,31]. The third insertional motif sequence was “GGGCCGAACCAGA (X-2)”, and the origin of X-2 was not identified by the BLAST search. The fourth insertional motif sequence was a complementary sequence of part of box α in HBV enhancer II. Similar to the HNF1 binding site, box α in HBV enhancer II regulates the transcription of the HBV genome. Previous experimental data have shown that box α modulates BCP activity and up-regulates BCP activity by more than 100-fold. As a canonical form of SV, the duplication of box α was observed in kidney transplantation recipients [50]. In some clinical studies, box α mutation was observed in patients with HCC [55,56,57,58,59,60,61,62]. These data elucidated the existence of insertional motif sequences, and also suggested their virological and clinical significance.

## 7. Classification of Complex SVs in HBV

In comparison with other genetic changes in HBV, complex SVs show more diverse patterns. In a previous report in mice, the pattern of complex SVs was categorized and their frequency was determined [23]. A total of 70 HBV strains with complex SVs were analyzed based on their SV patterns in order to reveal the combination of the highest frequency and to determine the number of patterns of complex SVs. They were categorized into six groups (class I, insertion (+)/deletion (+); class II, insertion (+)/deletion (+)/duplication (+); class III, insertion (+)/duplication (+); class IV, deletion (+)/duplication (+); class V, multiple duplications; class VI, highly complicated, which consisted of four or more SVs, as shown in Table 1. In addition, insertions were divided into five types (A to E).

The replication competence of HBV strains with complex SVs has been shown in some strains in previous studies. For example, the longitudinal time course of one strain was analyzed. At first, wild-type HBV genome was predominant, then the proportion of the strain with complex SVs increased and became the major strain [29].

Class I pattern had the highest frequency (70.0%). In addition, HNF1 binding site insertion was observed more frequently than for the others in class I. A total of 24/70 (34.3%) of the HBV strains with complex SVs were of the class I pattern with HNF1 binding site insertion. Complex SVs with the HNF1 binding site insertion were limited to HBV genotype A to E [31]. n, number.

## 8. SV Polymorphisms

### 8.1. SV Polymorphisms

Polymorphic SVs have been described in HBV research in the past. A 6 nt insertion in the core region specific to HBV/A has been described [6,7,63]. In the case of HBV genotype G, a unique partial genome sequence, which contained 36 nt insertion in the core region, was reported 10 years before HBV genotype G was defined [64,65,66]. It is now known that the 36 nt insertion is specific to HBV genotype G. Concerning SVs in the pre-S1 region, similarities of HBV/D and non-human primate HBVs with those of HBV/E and HBV/G have been identified [67,68].

### 8.2. SV Polymorphisms in the Core Region

**Figure 2:**
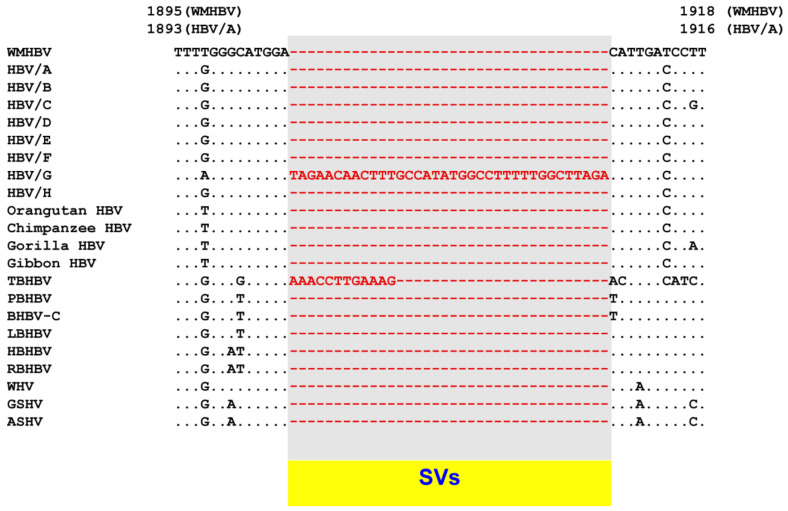
Alignment of the core ORF from nt 1895 to 1918 (WMHBV) showed that human HBV genotype G (HBV/G) and tent-making bat HBV (TBHBV) possess different SVs in this region. Reproduced from Reference [69], and modified. The consensus genome sequences of human HBV were determined using 150 HBV genotype A strains, 40 HBV genotype B strains, 168 HBV genotype C strains, 79 HBV genotype D strains, 38 HBV genotype E strains, 38 HBV genotype F strains, 13 HBV genotype G strains and 30 HBV genotype H. For non-human primates HBV, 8 orangutan HBV strains, 27 chimpanzee HBV strains, 6 gorilla HBV strains and 27 gibbon HBV strains were used. For bat HBV, 4 TBHBV strains, 3 Pomona bat HBV (PBHBV) strains, 3 unspecified bat HBV from China (BHBV-C) strains, 3 long-fingered bat HBV (LBHBV) strains, 1 horseshoe bat HBV (HBHBV) strain and 4 roundleaf bat HBV (RBHBV) strains were used. For rodent HBV, 14 woodchuck hepatitis virus (WHV) strains, 1 ground squirrel hepatitis virus (GSHV) strain and 1 arctic squirrel hepatitis virus (ASHV) were used. Information on the genetic sequences of human and non-human primate, bat and rodent HBVs are shown in Reference [69].

Analysis of complex SVs in human HBVs has shown that hidden genetic alterations can be elucidated through detailed bioinformatical analyses. The cause of the genetic diversity of different species of orthohepadnaviruses was investigated using techniques and experience obtained from the analysis of complex SVs. Pairwise and multiple alignment comparison among human HBV, non-human primate HBVs, bat HBVs and rodent HBV has shown that tent-making bat HBV (TBHBV) possesses a 12 nt insertion in the same region as human HBV genotype G (Figure 2) [69].

**Figure 3:**
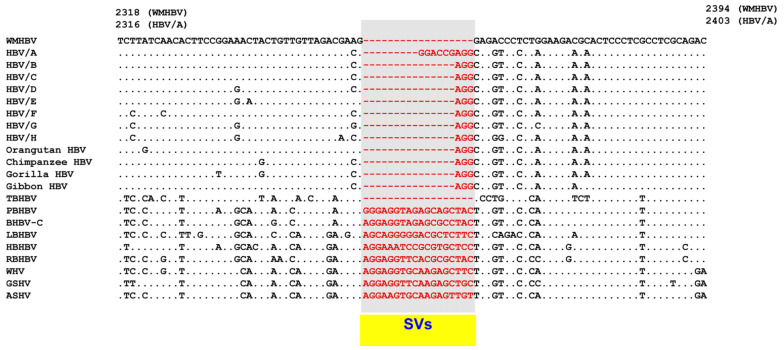
Alignment of the core ORF from nt 2318 to 2394 (WMHBV) showed different SVs in HBV/A and species of orthohepadnaviruses in this region. The polymorphic SVs change the length of amino acid residues in N-terminal helices of HBV polymerase terminal protein. Reproduced from reference 69, and modified. Details of the consensus genome sequences of human HBV and the non-human primate, bat and rodent HBV strains are provided in the legend to Figure 2. Genetic sequences of the strains are available in Reference [69].

In addition, in the core region, where human HBV genotype A has a 6 nt insertion, different SVs were observed [69], as shown in Figure 3. Up to a certain point, genetic differences were caused by the accumulation of point mutations. However, at some point, the nucleotide sequences were split into several particular patterns. These SVs were observed as polymorphic changes. These SVs were provisionally referred to as “SV polymorphisms” [69].

### 8.3. SV Polymorphisms in the Pre-S1 Region

**Figure 4:**
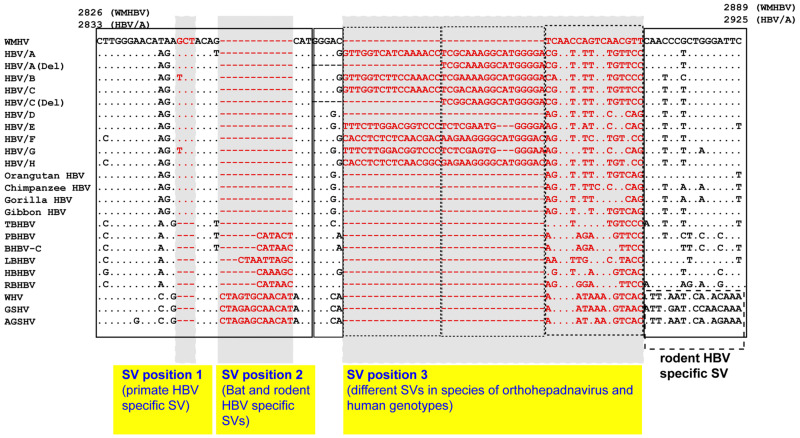
Alignment of the Pre-S1 ORF start site from nt 2826 to 2889 (WMHBV) showed different SVs in species with orthohepadnaviruses. Since multiple segments with SVs were observed in this region, the positions of SVs were designated as SV position 1 to 3. The SV polymorphisms in SV positions 1–3 change the length of amino acid residues of HBV polymerase spacer domain. Reproduced from reference 69, and modified. Details of the consensus genome sequences of human HBV and the non-human primate, bat and rodent HBV strains are provided in the legend to Figure 2. Genetic sequences of the strains are available in Reference [69].

In a previous study, SV polymorphisms of human HBV genotypes A to H were identified in the Pre-S1 region [30]. Subsequent analyses focused on Pre-S1 SVs in bat and rodent (woodchuck and squirrel) HBVs. Primate-, bat- and rodent-specific SVs have all been observed in this area (Figure 4) [69].

### 8.4. Clinical and Scientific Perspectives on Complex SVs and SV Polymorphisms

Among 70 HBV strains with complex SVs, at least two strains were recovered from patients with fulminant hepatitis, from two patients with severe chronic liver disease and 11 patients with HCC, respectively. The positions of complex SVs in the HBV genome and the types of insertional motifs determine the virologic characteristics and clinical manifestation of HBV strains with complex SVs. A previous article reported the transcriptional up-regulation of the viral genome by HNF1 binding site insertion, as well as the excessive production of viral protein and the accumulation of protein in the infected hepatocytes [29], data which suggest a potential risk of fulminant hepatitis or severe chronic liver disease.

In human and mouse genomes, DNA replication forks and template switching/microhomology-mediated break-induction replication can generate complex SVs [22]. The HBV polymerase has reverse transcriptase activity. In the replication step, the HBV relaxed circular DNA genome is converted to covalently closed circular DNA and dsl DNA, which is produced as a by-product of HBV replication, and is integrated at low rate into host chromosomal DNA [37]. Although detailed mechanisms such as the use of viral or cellular polymerase are not clear, the formation of complex SVs in HBV could be regulated by a similar mechanism to that found in eukaryotic genomes.

As for SV polymorphisms observed in orthohepadnavirus, hidden genetic changes in different species of orthohepadnavirus have been clarified, and these could be a key factor for the ability of hepadnavirus to infect a wide variety of hosts. Concerning polymorphic SVs in human HBV, experimental data have shown their role in viral genome replication and viral protein expression. Previous experimental data have shown that removing an insertional SV of 36 nt length in HBV/G caused a reduction in both the core protein expression and suppression of genome replication [70]. In addition, a recent study by Murayama and colleagues [71] showed that removing a 33 nt SV polymorphism in HBV/C, which changed the HBV/C construct in part of the pre-S1 region into SV polymorphisms in HBV/D and primate HBVs, caused a 10-times higher replication of the viral genome as compared with a wild-type HBV/C construct. Similar data were reported by Tong and colleagues [72]. Furthermore, SV polymorphisms in the pre-S region affected the production of large surface protein (LBS) and changed the subcellular distribution of LBS [73]. These data demonstrate the important role of SV polymorphisms observed in each of the human HBV genotypes.

## 9. Conclusions

Novel genetic alterations, referred to as complex SVs, in HBV have been identified, and these rearrangements alter the viral genome along with CP mutations (A1762T/G1764A), pre-core mutations, intergenotypic recombinations and many nucleotide substitutions. The characteristics of complex SVs were elucidated from analyses of 70 HBV strains with complex SVs. In addition, the existence of SV polymorphisms was demonstrated based on techniques used for analyses of complex SVs. SV polymorphisms may be related to the genetic diversity of orthohepadnaviruses. In addition, accumulating experimental data have shown the role of SV polymorphisms in human HBV genotypes. Further studies are needed to elucidate the virologic and clinical roles of SVs.

## Figures and Tables

**Table 1:** Classification of HBV strains with complex SVs.

Patterns	(*n* = 70)
**I. Insertion (+), Deletion (+)**	**49 (70.0%)**
Types of Insertion	
A. HNF1 binding site	24 (34.3%)
B. Insertion of unknown origin (X-1)	7 (10.0%)
C. Insertion of unknown origin (X-2)	3 (4.3%)
D. Sequence complementary to part of box α in enhancer II	6 (8.6%)
E. Miscellaneous	9 (12.9%)
II. Insertion (+), Deletion (+), Duplication (+)	6 (8.6%)
Types of Insertion	
A. HNF1 binding site	4 (5.7%)
B. Insertion of unknown origin (X-1)	1 (1.4%)
C. Insertion of unknown origin (X-2)	0 (0.0%)
D. Sequence complementary to part of box α in enhancer II	0 (0.0%)
E. Miscellaneous	1 (1.4%)
III. Insertion (+), Duplication (+)	5 (7.1%)
Types of Insertion	
A. HNF1 binding site	1 (2.9%)
B. Insertion of unknown origin (X-1)	2 (4.3%)
C. Insertion of unknown origin (X-2)	0 (0.0%)
D. Sequence complementary to part of box α in enhancer II	0 (0.0%)
E. Miscellaneous	0 (0.0%)
IV. Deletion (+), Duplication (+)	4 (5.7%)
V. Duplications (+)	1 (1.4%)
VI. Highly complicated (four or more SVs)	5 (7.1%)

Insertion patterns are shown in order of discovery. Some HBV strains with two SVs with an identical pattern and one other SV are included in group I, III and IV. SV, structural variation; HNF1, hepatocyte nuclear factor 1. Each of the 70 HBV strains were isolated from individual patients. Reproduced with permission of BMC Microbiology (Reference [31]).

## Data Availability

Not applicable.
